# CLINICOPATHOLOGIC EVALUATION OF MAMMARY PAGET'S DISEASE

**DOI:** 10.4103/0019-5154.39736

**Published:** 2008

**Authors:** Naser Tayyebi Meibodi, Vahid Mashayekhi Ghoyunlu, Zari Javidi, Yalda Nahidi

**Affiliations:** *From the Pathology Department Imam-Reza Educational Hospital, Mashhad University of Medical Sciences, Mashhad, Iran*; 1 *From the Dermatology, Department Imam-Reza Educational Hospital, Mashhad University of Medical Sciences, Mashhad, Iran*

**Keywords:** *Extramammary*, *intraepidermal neoplasia*, *mammary*, *Paget's disease*

## Abstract

Mammary and extramammary Paget's diseases are rare neoplasms of epidermis and mucosal epithelium. Due to their nonspecific and variable clinical view, they have differential diagnosis with eczema, melanoma, Bowen's disease, etc. To the best of our knowledge, no such study has been performed in Iran regarding the prevalence, clinical aspects, underlying disease and pathological characteristics of these two diseases. In this study, we have evaluated the clinical and histopathological aspects of this disorder.

**Materials and Methods:**

In this retrospective study, all Paget's biopsied samples referred to the Pathology Department of Imam-Reza hospital, Mashhad, since 1984 till 2004 were evaluated. Collected data were analyzed by descriptive statistical methods.

**Results:**

Among 98925 specimens, there were 29 cases of Paget's disease. All cases were married women suffering from mammary Paget. The mean age was 53 ± 11 years. Left and right breast involvement was observed in 17 and 12 cases, all unilateral. The most common clinical view was ulcerated (27%) and then erythematosus exudative plaques. More than 50% of patients were symptomatic. Most common symptoms were itching, pain and burning. The exclusive underlying pathological diagnosis was ductal carcinoma (55%).

**Discussion:**

In most cases, the clinical view of mammary Paget's disease was helpful. Unilateral ulcerated plaque was the most common clinical sign. Majority of the accompanying pathology was ductal carcinoma. We had no cases of extramammary Paget's disease in our study.

## Introduction

Paget's disease is an uncommon and relatively rare intraepithelial adenocarcinoma that presents clinically as ulcerated or excoriated plaques.^1^ Due to its benign clinical view, the diagnosis postpones for more than 6 months.^2^

Mammary Paget's disease (MPD) was discovered by James Paget in 1874 and extramammary type was found 15 years later by Radcliffe Crocker.^3^

MPD manifests as an underlying breast carcinoma^1^ that occurs in less than 5% of women suffering from breast cancer. It also may rarely occur in men.^4^

Extramammary Paget's disease (EMPD) is most often observed in areas that are rich in apocrine glands. Patients usually present with the eczematous crusted or excoriated gray white plaques.^3^ It may not or may be associated to underlying adnexal or visceral malignancies. Longtime follow-up and monitoring of the patient is necessary to rule out the disease relapse and diagnosis of underlying malignancies.^4^

To the best of our knowledge, since there is no study performed in Iran regarding the prevalence, clinical aspects, underlying disease and pathological characteristics of these two diseases, we have evaluated the clinical and histopathological aspects of this disorder in this study.

## Materials and Methods

In this descriptive retrospective study, all Paget's biopsied samples referred to Pathology Department of Imam-Reza hospital, Mashhad, since 1984 till 2004 were evaluated. Data collection was performed using questionnaires that consisted of questions regarding age, sex, site of involvement, underlying disease, underlying breast cancer, clinical findings and pathology reports. Collected data were analyzed by statistical methods such as Qui square, Kruskal-Wallis and Mann-Whitney tests using SPSS software (version 15).

## Results

In this 20-year study, all the 29 cases under this study were married women suffering from mammary Paget's disease. The mean patient age was 53 ± 11 years (ranging from 23 to 80 years). All MPDs were unilateral and more common in the left. The most common symptoms were itching (24.1%), pain (20.7%) and burning (6.9%); Among these cases, 48.3% were asymptomatic.

Ulcerated plaque (27.6%) and then exudative erythematosus plaque (24.3%), nodule on intact skin (24.2%), excoriated erythematosus plaque (10.3%), brown plaques (6.9%) and ulcerative erythematosus plaque (6.8%) were the main clinical manifestations.

The most prevalent diagnosis of the specimens was Paget's disease and breast cancer. Other differential diagnosis includes eczema, lichen simplex, psoriasis, candidiasis and morphea.

Among our cases, 55.2% had an underlying breast carcinoma. There was significant relationship between clinical manifestations and underlying disease (*P* = 0.001). All the nodular cases were associated with underlying ductal carcinoma.

## Discussion

Paget's disease is an uncommon form of epithelial adenocarcinoma that involves mammary and extramammary tissues.^1^ In both types of this disease, the clinical characteristics are the same and similar to infectious and inflammatory diseases.^5^ Mammary Paget is a rare disorder of the nipple-areola complex that is often associated with underlying *in situ* or invasive breast carcinoma.^2^,^6^,^7^ Accompanying of EMPD to underlying malignancy of skin or viscera is rare.^7^ Mammary Paget's disease (MPD) forms 0.5-4% of breast cancers and presents with progressive eczematous changes of areola, itching, resistant pain or burning and ulceration.^2^,^6^ There are two theories on Paget's histogenesis. The epidermotropic theory suggests that Paget's cells are ductal carcinoma cells that have migrated from an underlying carcinoma of the breast parenchyma to the epidermis of the nipple. The *in situ* transformation theory has been proposed to explain the development of this disorder in patients who does not have underlying mammary carcinoma or when they have underlying carcinoma anatomically remote from the nipple-areola complex. Paget's cells are believed to arise as malignant cells in the epidermis of the nipple independent from any other pathologic process within the breast parenchyma.^2^,^6^,^8^,^9^

Eczematoid changes of the nipple-areola complex and persisting soreness or itching, without obvious reason, is a suspicious symptom for Paget's disease of the breast and calls for thorough evaluation, including mammography. Exfoliative cytology and surgical biopsy according to the diagnostic standard may be useful.^6^

Microscopic view shows the infiltration of the epidermis by a large number of large malignant round and oval vesicular cells with pale or acidophil cytoplasm, large vesicluate or hyperchromatous nuclei and without intercellular bridges. The cytoplasm may contain periodic acid-Schiff (PAS)-positive, diastase-resistant granules, indicating the presence of neutral mucopolysaccharides. Rarely, acid mucopolysaccharides (sialomucin) may be identified by Alcian blue reaction at pH 2.5. Usually, basal keratinocytes lie between the Paget cells and the papillary dermis.^5^,^10^

Positive MUC1 and low-molecular-weight cytokeratins (CK) in conjunction with immunonegativity for high-molecular-weight CKs are the most diagnostically useful immunohistochemistry markers for mammary Paget.^5^

Although there are few articles in which MPD is reported in men, all of our 29 cases were women.^10^,^11^ The mean age of our cases was 53 years (ranging from 32 to 80 years), as supported by Kau's report in which the age average was 55 years (24-84 years).^10^ In our study, all MPDs were unilateral and more prevalent at the left side, but not significantly. In other similar studies, majority of MPDs were unilateral and not predominant in left or right side. Bilateral MPD is rarely reported.^10^

Similar to literatures, majority of our patients were asymptomatic (48.3%). Among symptomatic cases, itching (24.1%), pain (20.7%) and burning (7%) were the most common symptoms.^10^,^12^ Unlike to our study, Sakorafas reported bloody discharge in 37% of his MPD cases.^13^ In a study by Kazumi and colleagues on 104 MPD patients, nipple discharge and eczematous changes of nipple-areola complex were the most common findings.^14^ Plaques were the most prevalent clinical view of our patients (69%); this was similar to other reports in which erythematosus scaly or crusted plaques were the most common manifestations.^10^ In this study, the second clinical view were nodule (31%). Nodule prevalence is reported between 22 and 50% in the literature.^12^,^13^

We found underlying carcinoma in 55.2% cases. This was 98% in Kao series and 82-92% in Lioyd cases.^5^ Further, findings by Kawase showed underlying invasive and noninvasive cancer in 93.2% cases.^15^ Yim and colleagues performed a retrospective study on 38 MPD patients in which *in situ* or invasive ductal carcinoma mostly multifocal (73%) was found in 92% of cases even when no palpable mass was evident (85%).^15^ In 50% of cases considered by Harefuah, a palpable mass was evident and it usually represented an infiltrating carcinoma that involved axillary lymph nodes.^2^ These differences between our findings and mentioned studies, can be due to differences in follow-up by the patients in other medical centers, inadequate samples for diagnosis of underlying lesion, inappropriate follow-up, non-consent patients to perform biopsy or mastectomy in cases with non-palpable masses or intraepithelial *in situ* malignant transformation theory of Paget cells.

In this study no significant relationship between clinical symptoms and underlying disorder was detected. In MPD cases without underlying disease, no nodule was found and plaques were more common. However, in cases with underlying ductal carcinoma, nodule has a greater prevalence. Clinically, differential diagnosis for MPD includes nipple eczema, nipple erosive adenoma, Bowen's disease, superficial basal cell carcinoma, tinea, candidiasis and contact dermatitis.^12^,^16^ Moreover, in our study, diffe rential diagnosis included eczema, lichen simplex, psoriasis, morphea and candidiasis. MPD prognosis is associated with the nature of underlying tumor.^2^,^8^ Patients less than 60 years of age at diagnosis (stage II) involved lymph nodes, underlying infiltrative carcinoma, palpable mass or multifocal lesions have lower prognosis.^2^,^14^,^15^ Mastectomy is known as standard technique for MPD treatment. Nowadays, modern approaches are used to preserve the breast.^2^,^6^,^8^

## Conclusion

All cases in our study were mammary Paget's cases and ductal carcinoma was the most prevalent accompanying disorder. Therefore, if unilateral ulcerated plaques on nipple-areola complex suggest MPD, breast examination, mammography and even biopsy are highly recommended for ruling out the possible underlying carcinoma.
